# The difference in the association between included ECPR patients and neurological outcomes

**DOI:** 10.1186/s13054-023-04302-2

**Published:** 2023-01-25

**Authors:** Toru Hifumi, Akihiko Inoue, Norio Otani

**Affiliations:** 1 Department of Emergency and Critical Care Medicine, St. Luke’s International Hospital, 9-1 Akashi-cho, Chuo-ku, Tokyo, 104-8560 Japan; 2 Department of Emergency and Critical Care Medicine, Hyogo Emergency Medical Center, Kobe, Japan

**Keywords:** Extracorporeal cardiopulmonary resuscitation, Out-of-hospital cardiac arrest, Neurological outcome, Survival rate, Cardiac, Targeted temperature management

Dear Sir,

Watanabe et al. examined the impact of different targeted temperatures on the neurological outcomes for patients with out-of-hospital cardiac arrest (OHCA). Data were retrieved from the Japanese Association for Acute Medicine (JAAM)-OHCA registry, a prospective database in Japan. The patients received targeted temperature management (TTM) after undergoing extracorporeal cardiopulmonary resuscitation (ECPR). The study contained two groups of patients: those receiving normothermic TTM (n-TTM) (35–36 °C) and those receiving hypothermic TTM (h-TTM) (32–34 °C). No differences in 30-day favorable neurological outcomes [OR (95% CI) 1.01 (0.67–1.54)] or survival [OR (95% CI) 1.05 (0.76–1.46)] were observed between the two groups [1].

In Japan, two studies on the use of ECPR for OHCA have recently been reported. The first is a retrospective observational study (*N* = 890, 2014–2019) that used the JAAM-OHCA registry and included all kinds of OHCA patients [1]. The second is a retrospective registry study (*N* = 1644, 2013–2018) with the largest number of ECPR patients in the world; it is known as the Study of Advanced life support for Ventricular fibrillation with Extracorporeal circulation in Japan (SAVE-J II) [2].

Regarding the use of TTM after ECPR in Japan, the principal philosophy was speculated from a questionnaire survey distributed to medical institutions that participated in the SAVE-J II study [3]. According to the survey results, the targeted temperature of 33–34 °C was initiated in 83.3% of institutions as a first-line therapy. In contrast, the targeted temperature of 35–36 °C was initiated in only 11.1% of institutions. Thus, in Japan, especially before the results of TTM2 trial were published in 2021 [2], hypothermia at 33–34 °C was the main targeted temperature, and normothermia was supposed to be initiated only for patients with severe conditions such as hemodynamic instability, bleeding complications, and highly predicted poor prognosis comprehensively evaluated by physicians. Because of the uncontrolled bias (physician’s decision), it is highly suspected that hypothermia is associated with favorable neurological outcomes compared with normothermia even when bias was well controlled. On the basis of the SAVE-J II trial, we are now preparing for a detailed analysis of the association between the difference in targeted temperatures in OHCA patients receiving ECPR and the neurological outcomes.

The difference observed between the JAAM-OHCA registry (the current study) and SAVE-J II is mainly due to the difference in the included patients. The first included OHCA patients with all causes of arrest [1], whereas noncardiac conditions were excluded in the second [2]. According to another study using the JAAM-OHCA registry, the noncardiac cause was estimated to account for ~ 50% of cases [4]. However, noncardiac conditions, such as acute aortic dissection/aortic aneurysm, primary hypothermia, primary cerebral disorder, infection, drug intoxication, trauma, suffocation, and drowning, have different clinical outcomes and targeted temperatures [5]. Cardiac or noncardiac patients can almost be identified at the initiation of TTM; therefore, the results of the subgroup analysis may have contributed to the clinical decision for the targeted temperature.

## Data Availability

None.

## Abbreviations

OHCA: Out-of-hospital cardiac arrest
JAAM: Japanese Association for Acute Medicine
TTM: Targeted temperature management
ECPR: Extracorporeal cardiopulmonary resuscitation
SAVE-J: Study of Advanced life support for Ventricular fibrillation with Extracorporeal circulation in Japan
